# Supplementary material for: Role of the Ketogenic Diet Therapy and ACTH as Second Treatments in Drug-Resistant Infantile Epileptic Spasms Syndrome
Source: Nutrients. 2025 Jun 23;17(13):2085. doi: 10.3390/nu17132085 (PMC12251253; doi:10.3390/nu17132085)
Supplement: Supplementary file 1 [file nutrients-17-02085-s001.zip › nutrients-3685561-supplementary.pdf]

| Patients switched after initial treatment | **After KDT N=32** | **After ACTH N=35** | **P-Value** |
|---|---|---|---|
| Female * | 15 (47%) | 13 (37%) | 0.420 |
| Etiology known * | 22 (69%) | 27 (77%) | 0.439 |
| Age at epilepsy onset (months) ** | 3.6 (0-22) | 3.0 (0-27) | 0.990 |
| Time from epilepsy onset to trial treatment (days) ** | 128 (7-885) | 183 (7-504) | 0.231 |
| Drug-naïve at study inclusion | 6 (15%) | 5 (14%) | 0.622 |
| Number of ASMs before trial ** | 2 (0-7) | 2 (0-7) | 0.980 |
| Concomitant Vigabatrin * | 12 (38%) | 22 (63%) | 0.038 |
| Psychomotor development age-appropriate at baseline * | 1 (3%) | 1 (3%) | 0.507 |

**Supplemental Table S1. Baseline characteristics of patients switched after initial treatment**
* n (%), ** median, minimum-maximum. Data are displayed by initial treatment allocation for patients entering the second treatment plan (Initial KDT N=32; initial ACTH N=35). No relevant differences between the initial treatment allocations were observed.

| | WITHOUT VGB | | WITH VGB | |
|---|---|---|---|---|
| FIRST TREATMENT PHASE | KDT N=30 | ACTH N=20 | KDT N= 23 | ACTH N=28 |
| | 10/30 (33%) | 7/20 (35%) | 11/23 (48%) | 6/28 (21%) |
| SECOND TREATMENT PHASE | ACTH | KDT | ACTH | KDT |
| | 3/11 (27%) | 2/5 (40%) | 0/5 (0%) | 10/18 (56%) |
| RESCUE TREATMENT | ASM | ASM | ASM | ASM |
| | 3/8 (38%) | 1/3 (33%) | 2/5 (40%) | 1/8 (13%) |

**Supplemental Table S2. Seizure freedom per treatment phase without or with concomitant Vigabatrin treatment.**

n/N (%). VGB (Vigabatrin)

In infants without Vigabatrin (VGB) treatment, of 33 who did not respond to the first treatment phase, 17 infants were not switched to per protocol second treatment (ACTH or KDT), of whom 14 received second ASM-therapy and 3 epilepsy surgery (data not shown). In infants with VGB treatment, of 34 not responding to the first treatment phase, 11 infants were not switched to per protocol second treatment, of whom 9 received second ASM-therapy and 2 epilepsy surgery (data not shown).

| | WITHOUT VGB | | WITH VGB | |
|---|---|---|---|---|
| FIRST TREATMENT PHASE | KDT N=30 | ACTH N=20 | KDT N= 23 | ACTH N=28 |
| | 10/30 (33%) | 7/20 (35%) | 11/23 (48%) | 6/28 (21%) |
| SECOND TREATMENT PHASE | KDT-ACTH | ACTH-KDT | KDT-ACTH | ACTH-KDT |
| | 13/21 (62%) | 9/12 (75%) | 11/16 (69%) | 16/24 (67%) |
| RESCUE TREATMENT | KDT-ACTH-ASM | ACTH-KDT-ASM | KDT-ACTH-ASM | ACTH-KDT-ASM |
| | 16/21 (76%) | 10/12 (83%) | 13/16 (81%) | 17/24 (71%) |
| INTENTION-TO-TREAT | KDT-ACTH-ASM | ACTH-KDT-ASM | KDT-ACTH-ASM | ACTH-KDT-ASM |
| | 16/30 (53%) | 10/20 (50%) | 13/23 (57%) | 17/28 (61%) |

**Supplemental Table S3. Cumulative outcome per treatment sequence without or with concomitant Vigabatrin treatment.**
n (%)

| EEG-Data<br>n=67 | Baseline | 1 month | 6 months | 12 months |
|---|---|---|---|---|
| **Hypsarrhythmia*** | 67 (100%) | 16 (23.9%) | 6 (8.9%) | 4 (6%) |
| **Epileptic discharges** | 67 (100%) | 62 (92.5%) | 50 (74.6%) | 35 (52.2%) |
| **Sleep spindels** | 14 (20.9%) | 46 (68.6%) | 48 (71.6%) | 52 (67.6%) |

**Supplemental Table S4. EEG - Data**
n (%), * Hypsarrhyhtmia was present in all patients, although discontinuous or incipient in n= 10 infants at baseline. Epileptic discharges remained after 12 months in half of the patients and sleep spindels were present in around two thirds.

| Adverse effects | KDT<br>N=56 |
|---|---|
| Carnitine deficiency* | 2 (3.6%) |
| Kidney stones* | 0 (0%) |
| Growth Deficit* | 4 (7.1%) |
| Weight Gain* | 1 (1.8%) |
| Weight loss* | 1 (1.8%) |
| High cholesterol* | 8 (14.3%) |
| High triglycerides* | 7 (7.5%) |
| Low fluid intake* | 18 (32.1%) |
| Infusion* | 5 (8.9%) |
| Vomiting* | 3 (5.3%) |
| High ketosis* | 8 (14.3%) |
| Hypoglycemia* | 0 (0%) |
| Food refusal* | 1 (1.8%) |
| Solid food refusal* | 4 (7.1%) |
| Obstipation* | 9 (16.1%) |
| Diarrhea* | 3 (5.4%) |
| Long term side effects* | 0 (0%) |
| Infection* | 3 (5.4%) |
| Cholecystolithiasis* | 0 (0%) |

**Supplemental Table S5. Adverse effects of KDT**

*n (%). KDT was administered in a total of n= 56 patients (including 32 patients of the first treatment with KDT who needed second treatment and 24 patients who received KDT as second treatment).

| Z-Scores | Baseline | 3 months | 12 months |
|---|---|---|---|
| **Weight*** | -0.23 (-3.64 - 4.68) | 0.28 (-5.48 - 2.81) | -0.15 (-3.35 - 2.73) |
| **Length*** | -0.21 (-9.72 - 7.91) | 0.29 (-6.35 - 3.83) | 0.10 (-2.99 - 3.67) |
| **BMI*** | -0.18 (-8.17 - 8.56) | -0.23 (-3.64 - 4.68) | -0.05 (-4.84 - 2.12) |

**Supplemental Table S6. Z-scores of Growth during KDT**
*median (minimum and maximum).

No major changes in growth z-scores were observed. Infants remained on their percentiles, lower in single cases due to the underlying aetiology (metabolic epileptic encephalopathies) and limited mobility, which we recently published (Maass et al. 2024).
